# Morphology And Morphometry of Human External Ear with Its Significance in Sex Determination and Stature Estimation - An Observational Study

**DOI:** 10.12688/f1000research.160629.2

**Published:** 2025-03-17

**Authors:** Shwetha Acharya, Chandni Gupta, Vikram Palimar, Sneha Guruprasad Kalthur, Purnima Adhikari

**Affiliations:** 1 Department of Anatomy, Katsurba Medical College, Manipal, Manipal Academy of Higher Education, Manipal, Karnataka, 576104, India; 2 Department of Forensic Medicine , Katsurba Medical College, Manipal, Manipal Academy of Higher Education, Manipal, Karnataka, 576104, India

**Keywords:** Morphology, Morphometry, Sex, External Ear, Regression Equation, Stature

## Abstract

**Background:**

The human ear is unique to individuals, and ear prints, like fingerprints, are discrete enough to distinguish identical twins. Therefore, the external ear could be used to identify both living and deceased individuals. Therefore, this study aimed to evaluate the stature and sexual dimorphism of the external ear using various morphometric parameters and morphological features for forensic identification.

**Methods:**

This study included 40 participants (20 males and 20 females). Participants were between the age of 18-25yrs. Measurements were taken with the subject’s head in the Frankfort horizontal plane. 18 measurements were taken like ear length and width, lobule height and width, concha length and width, tragus length, morphological ear length, helical mastoid distal length, thickness of ear lobe, auricular inclination angle, antihelical take off angle, concha mastoid angle, auricular index, lobular index, concha index, ear attachment length index and conchal bowl depth on external ear were taken and the morphological features like shape of ear, ear lobe, darwin’s tubercle and ear tragus, attachment of ear lobe and form of helix of both the right and left ears were noted. A digital Vernier caliper was used to measure all parameters. The angles were measured using a goniometer. Normal distribution was verified using the Shapiro–Wilk test. For normally distributed parameters, an independent t-test was used, and for non-normally distributed parameters, the Mann-Whitney U test was used to compare sexes. To compare the right and left parameters, independent t-tests (for normally distributed data) and Mann-Whitney U tests (for non-normally distributed data) were applied. Sex determination and stature estimation were performed using logistic regression analysis.

**Results:**

In males and females, the most common shape was oval, and the ear lobe was free. The most common shape of the ear lobe in males was arched, while in females, it was triangular. In males and females, the most common form of the helix was the concave marginal. In males, the most common shape of the tragus was round, whereas in females, it was long. In males and females, the most common shape of Darwin’s tubercle was enlarged. It was seen that the right lobe width showed perfect separation, indicating its potential as an extremely reliable predictor. It was noted that in females, the strongest correlation with height was with the ear inclination angle on both sides.

**Conclusion:**

The results of this study can help forensic anthropologists identify the sex and stature of a person from various ear measurements.

## Introduction

Personal identification implies a determination of individualism established on specified morphological criteria that are distinctive to that person. In the circumstance of skeletal remnants, identification is very complex and needs precise investigation of these remnants. ^1^ Human beings present a wide range of variations which are distinctive and facilitate differentiating a person from another. ^2^

In addition to DNA profiling, several morphological traits and biometric parameters have been used in forensic analyses to differentiate one individual from another. Certain structural attributes used for this purpose include fingerprints, facial traits, footprints and external ear. ^3– 5^

As earprints, like fingerprints, are distinct enough, even the human ear can be used to recognize identical twins as it is unique to individuals. ^6– 8^

External ear is the most definitive features of the human face, also referred as the pinna or auricle. ^2^ Many forensic anthropologists revealed that auricle plays a vital role in identification of gender, stature, age and ethnicity. ^9^ Additionally, the shape, location and measurement of the auricle are specific to everyone same as the fingerprint thus assisting its utilization in forensics. ^2^ Frequently, during a crime scene investigation ear inscriptions are typically found on gates and windows where a possible criminal has been hearing for likely assault. Therefore, such spots are gathered and assessed using saved records to determine a match with the accused. Therefore, earprints provide valuable forensic evidence. ^10^ When an accused person wears protective hand gloves, his fingerprints will not be available during those cases, and ear morphology and biometrics are frequently used. ^11^

Ear morphology and morphometry are more important than typical biometric attributes such as facial recognition because they are rarely affected by aging. However, it was not affected by the facial expression alterations. In addition, there is no effect of anxiety on the ear as it can happen in other traits such as retina and the iris. ^12, 13^ Hence, the ear is used widely as a forensic tool for individual identification purposes because of its permanency and distinctiveness in persons after birth to maturity. ^14^

It has been found that sex can be identified by ear measurements with up to 69.3% precision in male individuals and 72% in females. ^9^ Previous studies have shown that estimation of stature and sexual dimorphism can be done using morphologic parameters of the ear like ear length, width, ear lobular length and width. ^15, 16^

To date, no study has been conducted on human ear morphometry and morphology in the South Indian Population. Therefore, this study was conducted to estimate stature, assess sexual dimorphism, and racial specificity based on the morphometry and morphology of the external ear in the South Indian population and to derive a regression equation to predict sex and stature estimation for forensic identification.

## Methods

### Study type: Observational study

Institutional ethical approval was obtained from the Kasturba Medical College and Kasturba Hospital Institutional Ethics Committee on 31 ^st^ May 31, 2024, before starting the research. (IEC NO – IEC2 – 113/2024)

Written informed consent was obtained from the participants before collecting their data and for publication of their images as per the informed consent form template obtained from the Kasturba Medical College and Kasturba Hospital Institutional Ethics Committee.’.

We used the STROBE Reporting Guidelines for our study; a complete checklist is available under the Reporting Guidelines. ^17^

Study Period: The current research was carried out from June 2024 to October 2024.

Study location: Department of Anatomy.

Sample Size: 40. (sample size computation using effect size, d=0.5; significance level=0.05; power=0.8; sample size, n=40; i.e., 20 males and 20 females)

Study population: 18-25yrs.

Inclusion criteria:

• All participants who do not having ear any deformity
• Only young individuals of 18-25 yrs were included

Exclusion criteria:

• Subjects having ear deformity
• Subjects below 18 and above 25 were eliminated from the research

Detailed description of procedure/processes:

Measurements were taken with the subject’s head in the Frankfort horizontal plane.

**Figure 1.**
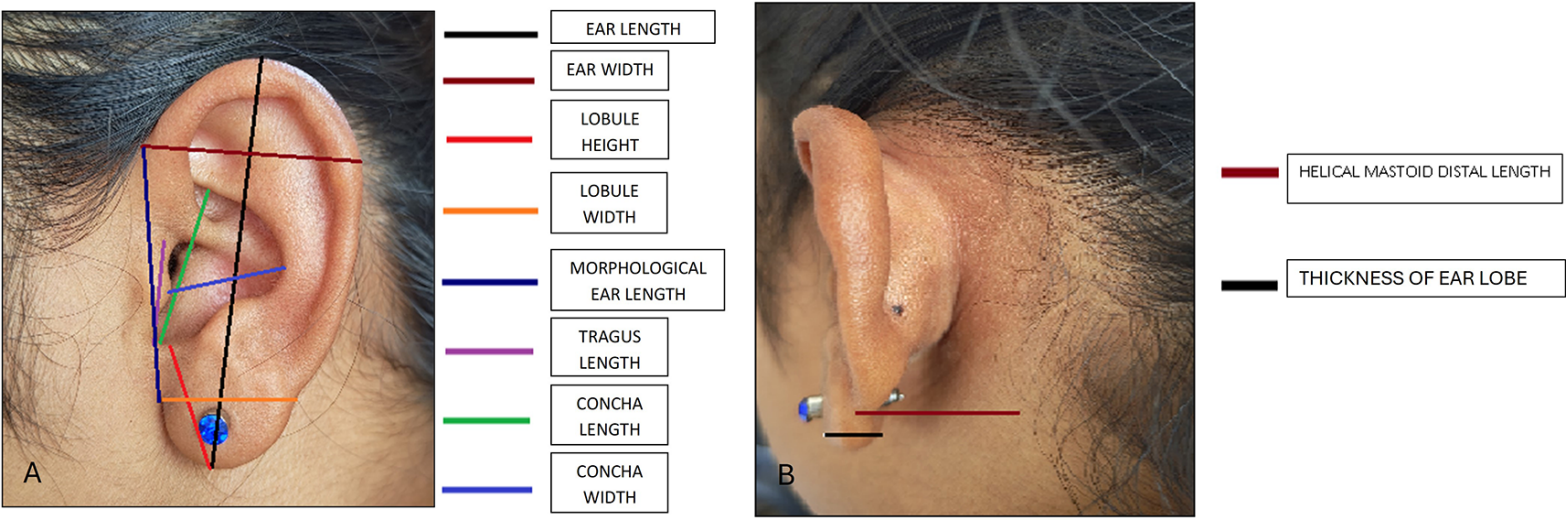
Linear measurements done on ear.

**Figure 2.**
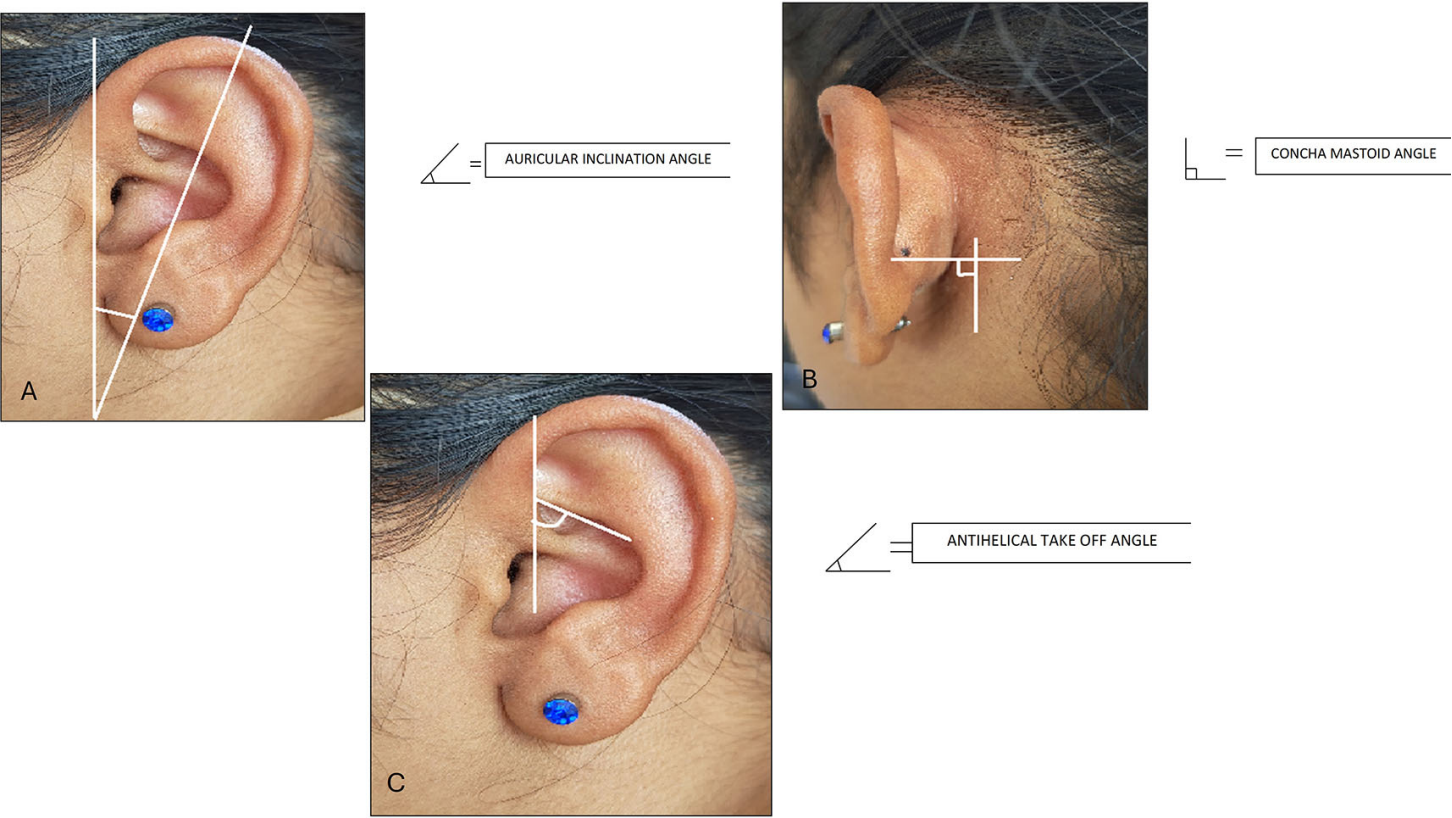
Angular measurements done on ear.

The following measurements were taken: ( Figures 1 and 2).

1) Ear length: It was determined as the distance between the most dependent part of the lobule to the superior end of the auricle.

2) Ear Width: It was determined as the distance between root of ear to helix where concavity is maximum.

3) Lobule height: It was measured as the distance from intertragic notch to most dependent part of the lobule.

4) Lobule width: It was determined as the maximal distance across the lobule taken transversely.

5) Concha length: It was determined as the distance from intertragic notch to the posterior aspect of tragus.

6) Concha width: It was determined as the distance from point where helix concavity is maximum to the posterior aspect of tragus.

7) Tragus length: Length between intertragic notch to tragion.

7) Morphological ear length: It was determined as the straight distance between otobasion superior and otobasion inferior.

8. Helical mastoid distal length: the distance from the mastoid process to the helix rim at the most posterior level of the superior auricular rim.

9. Thickness of ear lobe.

10. Auricular inclination angle: The angle measured between the length of the ear and the vertical.

11. Antihelical take off angle: The angle measured as the antihelix projects from the concha bowl.

12. Concha mastoid angle: The angle measured between the mastoid process and the concha bowl.

13. Auricular index = ear width ÷ ear length×100.

14. Lobular index = lobular width ÷ lobular length×100.

15. Concha index = concha width ÷ concha length×100.

16. Ear attachment length index = morphological ear length ÷ ear length×100.

18. Conchal bowl depth.

Tools used: A digital Vernier caliper with a precision of 0.001 mm was used to measure all parameters. The angles were measured using a goniometer. To avoid intra- and inter-observer bias, each measurement was performed twice by two people, and its average was recorded afterwards.

In addition to these morphometric measurements various morphological parameters were also noted down:

1. Ear Shape: Round, oval, triangular and rectangular.
2. Attachment of ear lobe: free, partially attached and fully attached.
3. Shape of ear lobe: arch, square, tongue and triangular.
4. Form of helix: rolled, wide, flat and concave marginal.
5. Shape of ear tragus: knob, round and long.
6. Shape of Darwin’s tubercle: projected, enlarged and nodosity.

### Statistical analysis

The data were evaluated using Jamovi 2.4 computer software. ^18^ Normal distribution was verified using the Shapiro–Wilk test. For normally distributed parameters, an independent t-test was used, and for non-normally distributed parameters, the Mann-Whitney U test was used to compare sexes. To compare the right and left parameters, independent t-tests (for normally distributed data) and Mann-Whitney U tests (for non-normally distributed data) were applied. Sex determination and stature estimation were performed using logistic regression analysis.

Underlying data is included in the Underlying Data section. ^19^

## Results

### Morphological parameters

Ear shape

**Figure 3.**
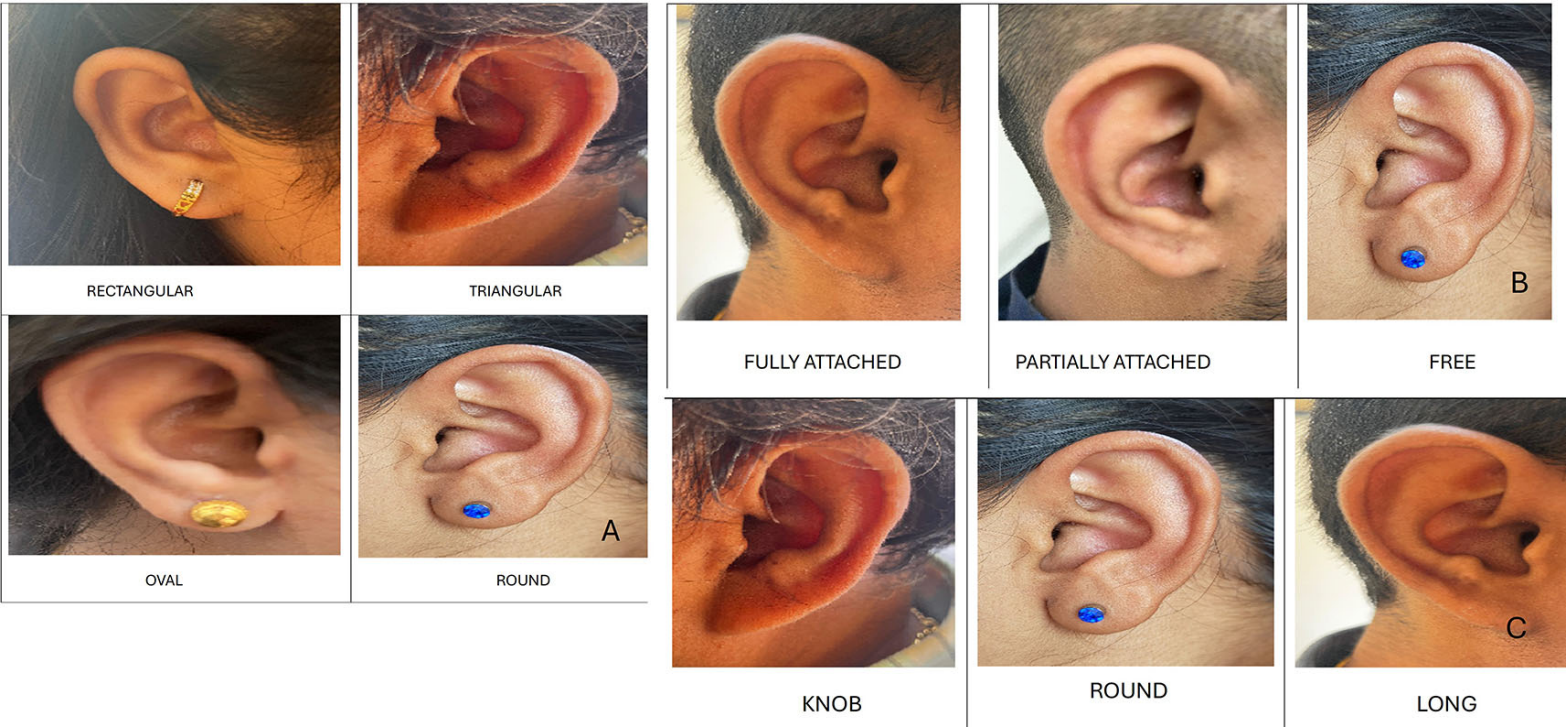
A. Shapes of ear. B. Attachments of ear lobe. C. Shapes of ear tragus.

In males, the most common shape was oval in 10 (50%), followed by triangular in 5 (25%), round in 3 (15%), and rectangular in 2 (10%). In females, the most common shape was oval 9 (45%), followed by triangular 5 (25%), round 4 (20%) and rectangular 2 (10%) ( Figure 3).

Attachment of ear lobe

In males, the most common ear lobe was free in 10 cases (50%), followed by partially attached in 7 (35%), and fully attached in 3 (15%) cases. In females, the most common ear lobe was free in 8 cases (40%), followed by partially attached and fully attached in 6 (30% each) cases ( Figure 3).

Shape of ear lobe

**Figure 4.**
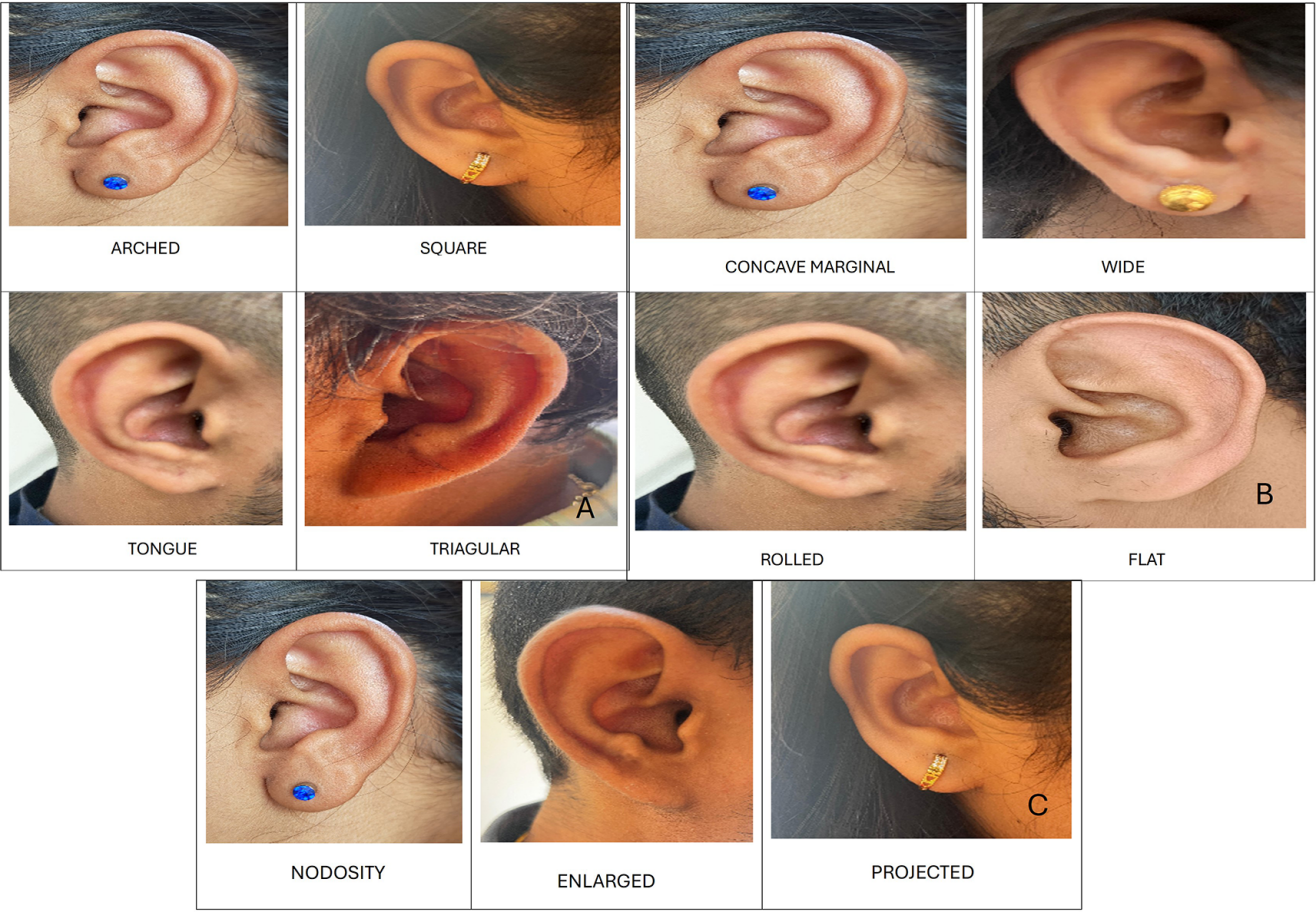
A. Shapes of ear lobe. B. Forms of helix. C. Shapes of Darwin’s tubercle.

In males, the most common shape was arched 10 (50%), followed by triangular 6 (30%), square 3 (15%), and tongue-shaped 1 (5%). In females, the most common shape was triangular (n = 7, 35%), followed by arched (n = 6, 30%), square (n = 5; 25%), and tongue-shaped (n = 2; 10%) ( Figure 4).

Form of helix

In males most common form of helix was concave marginal 9 (45%), followed by wide 5 (25%), flat and rolled 3 (15% each). In females most common form of helix was concave marginal 7 (35%), followed by flat 5 (25%), wide and rolled 4 (20% each) ( Figure 4).

Shape of ear tragus

In males, the most common tragus was round 12 (60%), followed by 5 (25%), and 3 (15%). In females, the most common shape of the tragus was long 9 (45%), followed by round 7 (35%), and knob shaped 4 (20%) ( Figure 3).

Shape of Darwin’s tubercle

In males, the most common shape of Darwin’s tubercle was enlarged by 10 (50%), followed by projected 8 (40%) and nodosity 2 (10%). In females, the most common shape of Darwin’s tubercle was enlarged in nine (45%), followed by projected seven (35%), and nodosity in four (20%) cases ( Figure 4).

Morphometric parameters

The descriptive statistics of all the parameters of the right and left sides were compared using the Mann-Whitney test (non-normally distributed data) and independent T Test (normally distributed data), as shown in Table 1.

**Table 1.** Descriptive statistics of right and left side with their comparison using Mann whitney test (non-normally distributed data) and independent T Test (normally distributed data).

Parameters		Mean ± SD	P value
Ear length (mm)	Right	63.2 ± 7.86	0.381
	Left	62.3 ± 7.77	
Ear width (mm)	Right	30.0 ± 2.85	0.411
	Left	29.4 ± 3.05	
Lobe height (mm)	Right	25.2 ± 2.20	0.038 *
	Left	24.2 ± 2.37	
Lobe width (mm)	Right	21.8 ± 4.21	0.758
	Left	21.6 ± 4.08	
Concha length (mm)	Right	14.5 ± 2.30	0.881
	Left	14.5 ± 2.12	
Concha width (mm)	Right	14.0 ± 1.78	0.860
	Left	14.0 ± 1.82	
Morphological ear length (mm)	Right	32.4 ± 2.78	0.299
	Left	31.8 ± 2.17	
Concha bowl depth (mm)	Right	11.3 ± 1.98	0.602
	Left	11.0 ± 1.88	
Helical mastoid distal length (mm)	Right	16.3 ± 1.44	0.099
	Left	15.7 ± 1.38	
Tragus length (mm)	Right	15.4 ± 2.40	0.405
	Left	15.1 ± 2.37	
Thickness of ear lobe (mm)	Right	7.14 ± 1.07	0.362
	Left	6.94 ± 0.91	
Auricular index	Right	48.0 ± 6.07	0.617
	Left	47.5 ± 5.71	
Lobular index	Right	87.3 ± 18.1	0.545
	Left	89.7 ± 17.4	
Concha index	Right	97.4 ± 9.50	0.989
	Left	97.4 ± 11.4	
Ear attachment length index	Right	185 ± 8.40	0.996
	Left	168 ± 7.33	
Ear inclination angle (°)	Right	15.1 ± 0.77	0.002 *
	Left	14.6 ± 0.81	
Antihelical take off angle (°)	Right	24.8 ± 1.43	0.045 *
	Left	24.2 ± 1.36	
Concha mastoid angle (°)	Right	88.7 ± 1.63	0.011 *
	Left	87.9 ± 1.65	

* P value < 0.05-Significant.

Comparing the parameters on the right and left sides, it was noticed that lobe height (p-value-0.038), ear inclination angle (p-value-0.002), antihelical takeoff angle (p-value-0.045), and concha mastoid angle (p-value-0.011) showed significant differences (p-value <0.05). The other parameters were not statistically significant.

The descriptive statistics of all the parameters of females with their comparison of the right and left sides using the Mann-Whitney test (non-normally distributed data) and independent T Test (normally distributed data) are shown in Table 2.

**Table 2.** Descriptive statistics of female parameters with their right and left comparison using Mann whitney test (non-normally distributed data) and independent T Test (normally distributed data).

Parameters	Mean± SD		
	Left	Right	P value
Ear length	68.4±2.26	69.5± 2.11	0.096
Ear width	30.9±2.67	31.2± 2.49	0.737
Lobe height	24.8± 2.39	25.2± 1.74	0.291
Lobe width	25.2±2.14	25.7± 1.99	0.515
Concha length	16.0± 1.79	16.3± 1.77	0.569
Concha width	14.5±2.04	15.1± 1.75	0.285
Morphological ear length	33.4±1.35	34.8± 1.36	0.002 *
Concha bowl depth	12.6±1.05	12.9±1.06	0.368
Helical mastoid distal length	15.2±1.48	15.8±1.72	0.236
Tragus length	15.5±1.57	16.0±1.78	0.357
Thickness of ear lobe	7.47±0.898	7.88± 0.878	0.152
Auricular index	45.2± 3.10	44.8± 3.00	0.718
Lobular index	103± 13.9	102±11.1	0.738
Concha index	90.7± 7.00	93.1±6.25	0.259
Ear attachment length	48.8 ±2.48	50.1± 2.86	0.326
Ear inclination angle	14.8± 0.927	15.4 ± 0.886	0.060
Anti helical take of angle	25.2± 0.834	25.8± 0.912	0.055
Concha mastoid angle	88.3± 1.45	88.8± 1.56	0.158
Ear length	68.4±2.26	69.5± 2.11	0.096
Ear width	30.9±2.67	31.2± 2.49	0.737
Lobe height	24.8± 2.39	25.2± 1.74	0.291
Lobe width	25.2±2.14	25.7± 1.99	0.515
Concha length	16.0± 1.79	16.3± 1.77	0.569
Concha width	14.5±2.04	15.1± 1.75	0.285
Morphological ear length	33.4±1.35	34.8± 1.36	0.002 *
Concha bowl depth	12.6±1.05	12.9±1.06	0.368
Helical mastoid distal length	15.2±1.48	15.8±1.72	0.236
Tragus length	15.5±1.57	16.0±1.78	0.357
Thickness of ear lobe	7.47±0.898	7.88± 0.878	0.152
Auricular index	45.2± 3.10	44.8± 3.00	0.718
Lobular index	103± 13.9	102±11.1	0.738
Concha index	90.7± 7.00	93.1±6.25	0.259
Ear attachment length	48.8 ±2.48	50.1± 2.86	0.326
Ear inclination angle	14.8± 0.927	15.4 ± 0.886	0.060
Anti helical take of angle	25.2± 0.834	25.8± 0.912	0.055
Concha mastoid angle	88.3± 1.45	88.8± 1.56	0.158

* P value < 0.05-Significant.

When comparing the parameters of females on the right and left sides, it was noticed that only the morphological ear length (p = 0.002) showed significant differences (p <0.05). The other parameters were not statistically significant.

The descriptive statistics of all the parameters of males with their comparison of the right and left sides using the Mann-Whitney test (non-normally distributed data) and independent T Test (normally distributed data) are shown in Table 3.

**Table 3.** Descriptive statistics of male parameters with their right and left comparison using Mann whitney test (non-normally distributed data) and independent T Test (normally distributed data).

Parameters	Mean± SD Median (Min-Max)		
	Left	Right	P value
Ear length	56.2±6.43	56.9 ± 6.13	0.758
Ear width	28.0 ±2.70	28.8± 2.72	0.348
Lobe height	23.7 ±2.28	25.2 ± 2.63	0.065
Lobe width	18.0±1.54	18.0± 1.19	0.933
Concha length	13.0±1.15	12.8 ± 1.17	0.787
Concha width	13.5±1.43	12.9± 0.938	0.190
Morphological ear length	30.3± 1.72	30.0± 1.49	0.617
Concha bowl depth	9.48± 1.04	9.62± 1.14	0.626
Helical mastoid distal length	16.3±1.01	16.8± 0.875	0.083
Tragus length	14.7± 2.96	14.8± 2.83	0.850
Thickness of ear lobe	6.41±0.575	6.41± 0.656	0.992
Auricular index	49.7±6.82	51.1± 6.77	0.253
Lobular index	76.6±8.17	72.1±8.17	0.093
Concha index	104± 11.1	102± 10.3	0.482
Ear attachment length	54.5± 6.82	53.4 ±6.53	0.626
Ear inclination angle	14.3± 0.615	14.9± 0.572	0.004 *
Anti helical take of angle	23.2± 1.01	23.9± 1.25	0.077
Concha mastoid angle	87.6± 1.82	88.6± 1.73	0.033 *

* P value < 0.05-Significant.

When comparing the parameters of males on the right and left sides, it was noticed that only the ear inclination angle (p = 0.004) and concha mastoid angle (p = 0.033) showed significant differences (p <0.05). The other parameters were not statistically significant.

A comparison of parameters between males and females is shown in Table 4. When comparing parameters between males and females, it can be noticed that lobe height right (p-value-0.970) and left (p-value-0.162), concha width left (p-value-0.071), tragus length right (p-value-0.135) and left (p-value-0.316), ear attachment length right (p-value-0.318) and left (p-value-0.335), ear inclination angle right (p-value-0.053) and left (p-value-0.059), concha mastoid angle right (p-value-0.753), and left (p-value-0.218) were not significantly different, and the rest of the parameters were significantly different (p-value <0.05).

**Table 4.** Comparison of male and female parameters using independent T-Test.

Parameters	P value
Ear length right	<.001 *
Ear length left	<.001 *
Ear width right	0.005 *
Ear width left	.001 *
Lobe height right	0.970
Lobe height left	0.162
Lobe width right	<.001 *
Lobe width left	<.001 *
Concha length right	<.001 *
Concha length left	<.001 *
Concha width right	<.001 *
Concha width left	0.071
Morphological ear length right	<.001 *
Morphological ear length left	<.001 *
Concha bowl depth right	<.001 *
Concha bowl depth left	<.001 *
Helical mastoid distal length right	0.028 *
Helical mastoid distal length left	0.006 *
Tragus length right	0.135
Tragus length left	0.316
Thickness of ear lobe right	<.001 *
Thickness of ear lobe left	<.001 *
Auricular index right	<.001 *
Auricular index left	0.010 *
Lobular index right	<.001 *
Lobular index left	<.001 *
Concha index right	0.003 *
Concha index left	<.001 *
Ear attachment length right	0.318
Ear attachment length left	0.335
Ear inclination angle right	0.053
Ear inclination angle left	0.059
Anti helical take of angle right	<.001 *
Anti helical take of angle left	<.001 *
Concha mastoid angle right	0.753
Concha mastoid angle left	0.218

* P value < 0.05-Significant.

Gender and ear dimension

Binomial logistic regression analyses were conducted to examine the correlations between various ear measurements and sex.

Multiple ear measurements were significant predictors of sex, as shown in Table 5.

1. Right ear length: (sex) = -59.889 + 0.916 × (right ear length)
2. Left ear length: (sex) = -45.564 + 0.710 × (left ear length)
3. Right lobe width: This predictor showed perfect separation, indicating extremely high predictive power
4. Right concha bowl depth: (gender) = -40.82 + 3.67 * (right concha bowl depth)
5. Left concha bowl depth: (gender) = -47.90 + 4.38 * (left concha bowl depth)

**Table 5.** Showing logistic regression analysis of ear morphometric predictors for gender.

Predictor	p value	Odds Ratio (95% CI)	Accuracy	AUC
Right ear length	<.001	2.50 (1.12-5.57)	0.925	0.988
Left ear length	<.001	2.03 (1.28-3.22)	0.925	0.978
Right lobe width	<.001	7.11e+11 (0.00 – Inf )	1.000	1.000
Right concha bowl depth	<.001	39.1 (1.49-1023)	0.975	0.985
Left concha bowl depth	<.001	80.2 (1.86-3464)	0.950	0.990

CI = confidence interval; AUC = area under the curve.

These results suggest that various ear measurements, particularly ear length, lobe width, and concha bowl depth, are strong predictors of sex. Right lobe width showed perfect separation, indicating its potential as an extremely reliable predictor.

### Stature and gender specific ear dimension

Female

Looking through the correlation matrices, the strongest correlation with height is with the ear inclination angle of both sides, with

• Right ear inclination angle: Pearson’s r = 0.489, (p-value-0.029)
• Left ear inclination angle: Pearson’s r = 0.599 (p-value-0.005)

Linear regression analyses were conducted to examine the correlation between the height and ear inclination angle.

There was a substantial positive correlation between height and the right and left ear inclination angles. The regression equations were as follows:

RightEarInclination Angle(in degrees)=7.72+1.40×(Height in feet)

LeftEarInclination Angle(in degrees)=5.02+1.80×(Height in feet)

The consistent finding of a significant positive correlation between height and ear inclination angle provides strong evidence of a solid association between these variables. The findings indicate that for every one-foot increase in height, the ear inclination angle increases by approximately 1.40 to 1.80 °.

Male

In males, there was no statistically significant correlation between height and any of the ear measurements. This suggests that in males ear dimensions may not be reliable for predicting a person’s height.

## Discussion

The pinna shape and dimensions are influenced by age, sex, and racial origin. ^2^ Some of the features of the external ear are unique and peculiar in that they resemble the fingerprint of a person. ^20^ Since the inclination, shape, and dimensions of the human external ear are very specific, they have been recognized as valuable anthropological variables for studying racial differences. ^21^

Rai et al. found that approximately 38.8% of male and 36.9% of females had attached earlobes. The most common shape of the external ear was oval in both male and females. The ear index was higher in the male ears, whereas the lobule index was higher in the female ears. In their study, there were substantial differences between males and females in the following parameters: ear length right and left, ear width right and left, concha width, length of right ear lobule, and lobule index. ^22^ In our study also most common ear shape was oval in both males and females. Fifteen% of male and 30% of females had attached ear lobes. The ear index in our study was higher in females while the lobule index was higher in males. This difference might be due to ethnic origin, as they conducted the study in North India and we conducted the study in South India. In our study, there were substantial differences between males and females in the following parameters: ear length right and left, ear width right and left, concha width, and lobule index, except right lobule length.

Kumari et al. found that in males the ear length, width, and lobule width of both ears were higher as compared to females. They also found by linear regression coefficient analysis that there was a strong association between right and left ear length and stature in females was. ^23^ In our study, all these parameters were greater in females than in males. A strong association between the right and left ear inclination angles and stature was observed in females using linear regression coefficient analysis. These differences in findings could be ethnic, as they have conducted research in North India, and we have conducted research in South India.

Hiware et al. found that statistically significant differences were observed between males and females in right ear height, left ear width, and width of the right and left ear lobe. ^24^ In our study, we also found statistically significant differences between these parameters in males and females.

Laxmi et al. found that the shape of the ear was oval in most specimens. The ear height and width were greater in males than females. The left auricular and lobular indices were higher in females than in males. Females had a longer right lobe than males. Tragus length was found to be greater in males than in females. ^25^ In our study, ear height and width were greater in females than in males. The left auricular index was higher in females than in males, but the lobular index was higher in males than in females. Right lobe length was the same in both sexes. Tragus length was found to be greater in females than in males. These differences in findings might be ethnic, as they have conducted research in the northwestern region of India, and we have conducted the study in South India.

Verma et al. found that free and attached ear lobes were noted in 35% and 65% of the cases, respectively. The oval ear shape was the most common, followed by triangular, rectangular, and round shapes. No statistical differences were noted in the ear and lobular indices between males and females. ^26^ In our study, 45% of free and 55% of attached ear lobes were observed. The oval ear shape was the most evident, followed by triangular, round, and rectangular. In our study, statistical differences were observed in the ear and lobular indices between males and females. These differences in findings can be ethnic, as they have conducted research in the northwestern and northeastern regions of India, and we have conducted the study in South India.

Farhan et al. found significant differences were noticed regarding gender in lobule height with respect to sex. Free lobules were noted in 66% of males compared to 54% of females. The most common ear shape was triangular in males and rectangular in females. ^27^ In our study, there was no significant difference noted regarding gender in lobule height between the sexes. In our study, 50% of male free lobules were noted compared to 40% of female free lobules. The most common shape was oval in both males and females. These differences in findings can be racial, as they have conducted research in Iraq, and we have conducted research in South India.

Bozkır et al. observed that all parameters were higher in males than in females. While in our study most parameters were higher in females as compared to males. ^28^ These differences in findings can be racial as they have conducted the research in Turkey, and we have conducted the research in South India.

Rani D et al. found that ear length, ear breadth, exhibited significant sex differences on both sides (p<0.05). Significant bilateral differences (p<0.05) were reported for all the measurements of the ear in males except for lobule height, whereas significant side differences (p<0.05) were shown for lobule width among females. ^29^ In our study also significant sex differences was noted on both sides in ear length (p<0.001 in each) and ear breadth (Right- p-0.005, left- p-.001). But there was no significant side differences noted in any of the parameters of the ear among males and females.

Khobkhun P et al. found that there was no significant difference in any of the auricular dimensions between the right and the left sides. They also found that the auricular length, auricular width, and conchal length were larger in male than in female participants. ^30^ While in our study we also found that that there was no significant difference in any of the auricular dimensions between the right and the left sides except lobe height (p-0.038). In our study all parameters related to auricle were larger in males as compared to females.

Boesoirie SF et al. found that auricle length, width, as well as concha length of men tended to be greater than women. However, the women’s lobule length was longer compared to men’s, while the lobule and concha width tended to be the same. ^31^ In our study in our study all parameters related to auricle were larger in males as compared to females. This might be due to racial differences as they have conducted the study in Sudanese population and the present study was conducted in Indian population.

Fakorede et al. found that oval ears were more common, followed by round ears. Arch-shaped lobules were the most frequently seen. Free earlobe attachment was the most common among the Nigerian populations, followed by partial attachment, while the attached earlobe was the least expressed. They also found that a knob-shaped tragus was the most prevalent. The wide form of the helix was the most evident. The nodosity-shaped Darwin tubercle appears to be the most prevalent. When comparing the right and left ear parameters, it was noted that all parameters were statistically significant, except for ear width. They also found that ear length, lobule height, lobule width, and concha length mainly contribute to sex classification. ^11^ In our study, the most common ear shape was oval and arch-shaped. Even earlobe attachment was free in our study, followed by partial and full attachment. In our study, round-shaped tragus and concave marginal form of the helix were mostly observed. An enlarged Darwin’s tubercle was mostly observed. Comparing the parameters on the right and left sides, it was noticed that lobe height (p-value- 0.038), ear inclination angle (p-value- 0.002), antihelical takeoff angle (p-value- 0.045), and concha mastoid angle (p-value- 0.011) showed significant differences (p-value <0.05). The other parameters were not statistically significant. In our study, ear length, lobe width, and concha bowl depth were strong predictors of gender. Some differences in findings can be racial, as they have conducted research in Nigeria, and we have conducted research in South India.

Morphological changes and morphometric parameters of the human ear can be utilized jointly with forensic DNA evaluation to solve complicated incidents, principally where fingerprints or facial recognition tools are not accessible. ^11^ The results of this study will help in identification of stature and sex from various ear parameters.

### Limitations of the study

• Small sample size.
• To predict male sex, no ear parameters were significant.

This study aimed to predict sex and stature based on ear dimensions. In both males and females, the common shape was oval, and the ear lobe was free. The most common shape of the ear lobe in males was arched, while in females, it was triangular. In males and females, the most common form of the helix was the concave marginal. In males, the most common shape of the tragus was round, whereas in females, it was long. In males and females, the most common shape of Darwin’s tubercle was enlarged.

Comparing the parameters on the right and left sides, it was noticed that lobe height, ear inclination angle, antihelical takeoff angle, and concha mastoid angle showed significant differences (p-value <0.05).

When comparing the parameters of females on the right and left sides, it was noticed that only the morphological ear length showed significant differences (p <0.05). When comparing the parameters of males on the right and left sides, it was noticed that only the ear inclination angle and concha mastoid angle showed significant differences (p <0.05).

When comparing parameters between males and females, it can be noticed that lobe height right and left, concha width left, tragus length right and left, ear attachment length right (and left, ear inclination angle right and left, concha mastoid angle right, and left were not significantly different (p-value >0.05).

It was seen that the right lobe width showed perfect separation, indicating its potential as an extremely reliable predictor. It was noted that in females, the strongest correlation with height was with the ear inclination angle on both sides.

## Ethical considerations

Institutional ethical approval was obtained from the Kasturba Medical College and Kasturba Hospital Institutional Ethics Committee on 31 ^st^ May 31, 2024, before starting the research on 5 ^th^ June 2024 (IEC NO – IEC2 – 113/2024).

## Data Availability

Morphology And Morphometry of Human External Ear with Its Significance in Sex Determination and Stature Estimation - An Observational Study. https://figshare.com/articles/dataset/Data_Excel_sheet/28120586?file=51439520. doi: 10.6084/m9.figshare.28120586. ^19^ The project contains the following underlying data: Data sheet of all 40 cases. The study contains the following extended data. Morphology And Morphometry of Human External Ear with Its Significance in Sex Determination and Stature Estimation - An Observational Study. https://doi.org/10.6084/m9.figshare.28137164.v5. ^32^ Data are available under the terms of the Creative Commons Zero “No rights reserved” data waiver (CC0 1.0 Public domain dedication). We used STROBE checklist for “Morphology And Morphometry of Human External Ear with Its Significance in Sex Determination and Stature Estimation - An Observational Study.” https://figshare.com/articles/online_resource/Strobe_checklist/28120598?file=51564029 doi: 10.6084/m9.figshare.28120598. ^17^ Data are available under the terms of the Creative Commons Zero “No rights reserved” data waiver (CC0 1.0 Public domain dedication).
