# Supplementary material for: Morphological And Morphometric analysis of Human External Ear with Its Implications in Sex and Stature Estimation -– A Preliminary Observational Study
Source: F1000Res. 2025 May 15;14:119. Originally published 2025 Jan 22. [Version 3] doi: 10.12688/f1000research.160629.3 (PMC12032520; doi:10.12688/f1000research.160629.3)
Supplement: Peer review history

# Reviewer report: Reviewer response for version 3

**DOI:** 10.5256/f1000research.181180.r385019

**Published:** 2025-06-04

**Authors:** Nurdan Sezgin

**Affiliations:** 1 Kütahya Health Sciences University, Kütahya, Turkey

The authors' revisions are deemed sufficient. There is no problem in indexing.

Is the work clearly and accurately presented and does it cite the current literature?

Partly

If applicable, is the statistical analysis and its interpretation appropriate?

Yes

Are all the source data underlying the results available to ensure full reproducibility?

Yes

Is the study design appropriate and is the work technically sound?

Yes

Are the conclusions drawn adequately supported by the results?

Partly

Are sufficient details of methods and analysis provided to allow replication by others?

Yes

Reviewer Expertise:

Forensic anthropology, identification, age and sex estimation from facial images and ear images

I confirm that I have read this submission and believe that I have an appropriate level of expertise to confirm that it is of an acceptable scientific standard.

# Reviewer report: Reviewer response for version 3

**DOI:** 10.5256/f1000research.181180.r385016

**Published:** 2025-05-29

**Authors:** Jolly Agarwal

**Affiliations:** 1 ANATOMY, Government doon medical college, Dehradun, Uttarakhand, India

No further comments

Is the work clearly and accurately presented and does it cite the current literature?

Partly

If applicable, is the statistical analysis and its interpretation appropriate?

Yes

Are all the source data underlying the results available to ensure full reproducibility?

Yes

Is the study design appropriate and is the work technically sound?

Yes

Are the conclusions drawn adequately supported by the results?

Yes

Are sufficient details of methods and analysis provided to allow replication by others?

Yes

Reviewer Expertise:

Anatomist, Osteology, Medical Education, Histology and Radiology

I confirm that I have read this submission and believe that I have an appropriate level of expertise to confirm that it is of an acceptable scientific standard.

# Reviewer report: Reviewer response for version 2

**DOI:** 10.5256/f1000research.179146.r371387

**Published:** 2025-04-29

**Authors:** Nurdan Sezgin

**Affiliations:** 1 Kütahya Health Sciences University, Kütahya, Turkey

There are some points that need attention in the study:

 1. The number of current publications in the references can be increased. Ear morphology studies have attracted more attention in recent years. Therefore, the number of publications after 2020 can be increased.

 2. It may be more meaningful to express measurements with anthropometric points (landmarks) when defining them. In fact, this marking can be done in the images.

 3. Working with 40 ear samples can be considered sufficient in terms of descriptive statistics. However, when comparing between sexes, the number is insufficient.

 4. A table is given regarding the statistical significance of ear measurements, but it is not given which measurement has which value in female and male. Including this information is important in the following respect: if the sex difference in ear measurements seems large, a sample group of 40 people can be considered sufficient. However, if the difference is small or at an average level, the number of samples are small (I am evaluating it for 40 people).

 5. Therefore increasing the sample size of the study and making a new statistical calculation will both take time and reduce the motivation of the researchers. So, the title of this study can be updated as follows: If the study will continue with a larger sample, "pilot study" should be added. If this study will remain with 40 people and a new, larger database will not be used, it is recommended to update it by adding "preliminary observational study".

Is the work clearly and accurately presented and does it cite the current literature?

Partly

If applicable, is the statistical analysis and its interpretation appropriate?

Yes

Are all the source data underlying the results available to ensure full reproducibility?

Yes

Is the study design appropriate and is the work technically sound?

Yes

Are the conclusions drawn adequately supported by the results?

Partly

Are sufficient details of methods and analysis provided to allow replication by others?

Yes

Reviewer Expertise:

Forensic anthropology, identification, age and sex estimation from facial images and ear images

I confirm that I have read this submission and believe that I have an appropriate level of expertise to confirm that it is of an acceptable scientific standard, however I have significant reservations, as outlined above.

# Response

**Published:** 2025-05-05

**Authors:** Chandni Gupta

**Affiliations:** Anatomy, Kasturba Medical college, Manipal, Manipal, Karnataka, India

1. The number of current publications in the references can be increased. Ear morphology studies have attracted more attention in recent years. Therefore, the number of publications after 2020 can be increased. -

**Response: **Done added.

 2. It may be more meaningful to express measurements with anthropometric points (landmarks) when defining them. In fact, this marking can be done in the images.

**Response**- Added the landmarks in the images.

 3. Working with 40 ear samples can be considered sufficient in terms of descriptive statistics. However, when comparing between sexes, the number is insufficient.

**Response**- This sample size we got from our statistics department by seeing from previous studies as we were doing both morphometric and morphological study.

 4. A table is given regarding the statistical significance of ear measurements, but it is not given which measurement has which value in female and male. Including this information is important in the following respect: if the sex difference in ear measurements seems large, a sample group of 40 people can be considered sufficient. However, if the difference is small or at an average level, the number of samples are small (I am evaluating it for 40 people).

**Response**- Added the values of measurement in table 4. And Sex difference is seen in all parameters except lobe height (right: p-value = 0.970, left: p-value = 0.162), concha width (left: p-value = 0.071), tragus length (right: p-value = 0.135, left: p-value = 0.316), ear attachment length (right: p-value = 0.318, left: p-value = 0.335), ear inclination angle (right: p-value = 0.053, left: p-value = 0.059), and concha mastoid angle (right: p-value = 0.753, left: p-value = 0.218). All other parameters were found to be significantly different, with p-values less than 0.05.

 5. Therefore increasing the sample size of the study and making a new statistical calculation will both take time and reduce the motivation of the researchers. So, the title of this study can be updated as follows: If the study will continue with a larger sample, "pilot study" should be added. If this study will remain with 40 people and a new, larger database will not be used, it is recommended to update it by adding "preliminary observational study".

**Response: **Added in title “Morphological And Morphometric analysis of Human External Ear with Its Implications in Sex and Stature Estimation -– A Preliminary Observational Study”

# Reviewer report: Reviewer response for version 2

**DOI:** 10.5256/f1000research.179146.r374367

**Published:** 2025-04-29

**Authors:** Anyanwu Godson Emeka

**Affiliations:** 1 Kampala International University, Ishaka, Uganda

The focus of this observational study was the morphology and morphometry of the human external ear studied in 40 young adults (20 males, 20 females) aged 18–25. Also studied was the reliability of generated data for sex determination and stature estimation in the scope of forensic purposes. The study used standardized measurements and morphological assessment of different ear features (lobe type, tragus shape, helix form, Darwin’s tubercle) and revealed significant sexual dimorphism and correlations to height, as well as the right ear lobe width and ear inclination angle as potential reliable predictors. The results indicate the forensic significance of external ear characteristics in personal identification.

1. Title as it is looks a bit clumsy, I suggest: Morphological and Morphometric Analysis of the Human External Ear: Implications for Sex and Stature Estimation
2. The two sentences in the background of the abstract are awkwardly phrased with some redundancy . The word “Therefore” is used twice in consecutive sentences, which is redundant , and this disrupts the flow. The awkward phrasing of the sentence … “. this study aimed to evaluate the  stature and sexual dimorphism of the external ear” is slightly misinforming because the ear is not attributed with stature and sexual dimophism, rather the stature and gender of individuals  can be estimated from its features.
3. i have problem with method part of the abstract, i feel the study design [eg cross sectional , longitudinal etc] and recruiting process [ eg simple random, purposive etc] would have been captured very briefly here. Suggestion “This was a cross-sectional observational study involving …….  recruited from  …………  using purposive sampling/ simple random technique.
4. The statement where you used the word “like” as captured in this sentence “18 measurements were taken like ear length and width, lobule height and width …..’ does not clearly connote you measured these parameters, you are indirectly saying you measured parameters that are similar to …. Pls recast sentence
5. In this sentence “A digital Vernier caliper was used to measure all parameters. The angles were measured using a goniometer. “ you said that a digital caliper was used to measure all parameters, when this is not correct, because the angles are part of the parameters studied and you measured them with goniometers. Pls recast sentence
6. I have concerns with the  current results paragraph because it lacks quantitative depth and clarity in highlighting statistical significance, distribution patterns, and key findings that strengthen forensic value.
7. Your conclusion is vague and lacks scope and specificity. **In your revision ** summarize which specific measurements were most useful (e.g., right earlobe width, ear inclination angle), as identified in your results.

 

1. The two sentences below are from your introduction, while one is in the present tense, the other is in the past. Pls recast to maintain consistency

 

1. This statement is an over-statement pls recast: “To date, no study has been conducted on human ear morphometry and morphology in the South Indian Population’

 

1. **Methods** section lacks coherence and feels more like a **list of procedures** than a well-structured narrative.  The section reads like disconnected bullet points rather than a continuous, organized description of the research process. Also the  narrative jumps between ethical clearance, reporting guidelines, study period, and sampling criteria without transition or thematic grouping.
2. The same observation made in 11 above is also noted in the discussion sections of this work

Is the work clearly and accurately presented and does it cite the current literature?

No

If applicable, is the statistical analysis and its interpretation appropriate?

Partly

Are all the source data underlying the results available to ensure full reproducibility?

Yes

Is the study design appropriate and is the work technically sound?

No

Are the conclusions drawn adequately supported by the results?

Partly

Are sufficient details of methods and analysis provided to allow replication by others?

Yes

Reviewer Expertise:

My study encompasses spine biomechanics, obesity-associated musculoskeletal problems, and anatomical risk determinants in orthopedics and neurodegenerative diseases. I am recognized for promoting forensic morphometry and anatomical teaching through creative tools, including the award-winning Anatomy Adventure board game.

I confirm that I have read this submission and believe that I have an appropriate level of expertise to state that I do not consider it to be of an acceptable scientific standard, for reasons outlined above.

# Response

**Published:** 2025-05-05

**Authors:** Chandni Gupta

**Affiliations:** Anatomy, Kasturba Medical college, Manipal, Manipal, Karnataka, India

1. Title as it is looks a bit clumsy, I suggest: Morphological and Morphometric Analysis of the Human External Ear: Implications for Sex and Stature Estimation- Changed the title
2. The two sentences in the background of the abstract are awkwardly phrased with some redundancy . The word “Therefore” is used twice in consecutive sentences, which is redundant , and this disrupts the flow. The awkward phrasing of the sentence … “. this study aimed to evaluate the  stature and sexual dimorphism of the external ear” is slightly misinforming because the ear is not attributed with stature and sexual dimophism, rather the stature and gender of individuals  can be estimated from its features.-Corrected the sentence.
3. i have problem with method part of the abstract, i feel the study design [eg cross sectional , longitudinal etc] and recruiting process [ eg simple random, purposive etc] would have been captured very briefly here. Suggestion “This was a cross-sectional observational study involving …….  recruited from  …………  using purposive sampling/ simple random technique.- Added
4. The statement where you used the word “like” as captured in this sentence “18 measurements were taken like ear length and width, lobule height and width …..’ does not clearly connote you measured these parameters, you are indirectly saying you measured parameters that are similar to …. Pls recast sentence- Changed
5. In this sentence “A digital Vernier caliper was used to measure all parameters. The angles were measured using a goniometer. “ you said that a digital caliper was used to measure all parameters, when this is not correct, because the angles are part of the parameters studied and you measured them with goniometers. Pls recast sentence- Corrected the sentence
6. I have concerns with the  current results paragraph because it lacks quantitative depth and clarity in highlighting statistical significance, distribution patterns, and key findings that strengthen forensic value.- Added
7. Your conclusion is vague and lacks scope and specificity. **In your revision ** summarize which specific measurements were most useful (e.g., right earlobe width, ear inclination angle), as identified in your results.- Added

 

1. The two sentences below are from your introduction, while one is in the present tense, the other is in the past. Pls recast to maintain consistency- Changed

 

1. This statement is an over-statement pls recast: “To date, no study has been conducted on human ear morphometry and morphology in the South Indian Population’- Changed

 

1. **Methods** section lacks coherence and feels more like a **list of procedures** than a well-structured narrative.  The section reads like disconnected bullet points rather than a continuous, organized description of the research process. Also the  narrative jumps between ethical clearance, reporting guidelines, study period, and sampling criteria without transition or thematic grouping.- This was made according to Strobe guidelines. And the template was giving from the F1000 journal, so we maintained it.
2. The same observation made in 11 above is also noted in the discussion sections of this work- Corrected the discussion and I have discussed the previous results with our study and added why they are similar or different from our study and added few more studies.

# Reviewer report: Reviewer response for version 2

**DOI:** 10.5256/f1000research.179146.r374370

**Published:** 2025-04-25

**Authors:** Adil Asghar

**Affiliations:** 1 All India Institute of Medical Sciences, Patna, India

1. **Clarify Limitation Regarding Generalizability**
  - **Issue**: Although “population” was removed from the title, the findings are still generalized in the text.
  - **Required Action**: Re-emphasize that results are applicable **only to young South Indian adults (18–25 years)** and may **not generalize across age, region, or ethnicity**.
2. **Address Small Sample Size in Interpretation**
  - **Issue**: With just 40 participants, some results—like "perfect separation" in logistic regression—might be artifacts of overfitting.
  - **Required Action**: Clearly state that **results are preliminary and exploratory**, and that **larger studies are needed** to validate these regression models and confirm predictors.
3. **Include AUC Curves or Graphs for Sex Prediction**
  - **Issue**: AUC values are reported, but no visual ROC curve or regression diagnostics are shown.
  - **Required Action**: Include **graphical output** of logistic regression model (e.g., ROC curve, classification plot) to visually support claims of predictive strength.

�� **Minor Revisions Suggested**

1. **Language and Grammar**
  - Rephrase awkward phrases:
    - “Subjects who do not having ear deformity” → “Subjects without any ear deformities.”
    - “Measurement was performed twice by two people” → “Each measurement was independently performed twice by two observers.”
  - Perform a **light grammar and clarity check** across the manuscript.
2. **Duplicate or Misplaced Text**
  - Table headers or descriptions appear to repeat on some pages (possibly due to PDF formatting).
  - Check for **repetitive statistical table text** and ensure **clean formatting** before indexing.
3. **Improve Figure Captions and Labels**
  - **Figures 1–4** could use more descriptive captions to be interpretable on their own.
  - Include **scale markers** where appropriate.

Is the work clearly and accurately presented and does it cite the current literature?

Yes

If applicable, is the statistical analysis and its interpretation appropriate?

Partly

Are all the source data underlying the results available to ensure full reproducibility?

Partly

Is the study design appropriate and is the work technically sound?

Yes

Are the conclusions drawn adequately supported by the results?

Partly

Are sufficient details of methods and analysis provided to allow replication by others?

Yes

Reviewer Expertise:

NA

I confirm that I have read this submission and believe that I have an appropriate level of expertise to confirm that it is of an acceptable scientific standard, however I have significant reservations, as outlined above.

# Response

**Published:** 2025-04-26

**Authors:** Chandni Gupta

**Affiliations:** Anatomy, Kasturba Medical college, Manipal, Manipal, Karnataka, India

1. **Clarify Limitation Regarding Generalizability**
  - **Issue**: Although “population” was removed from the title, the findings are still generalized in the text.
  - **Required Action**: Re-emphasize that results are applicable **only to young South Indian adults (18–25 years)** and may **not generalize across age, region, or ethnicity**- Added in the abstract, discussion and conclusion
2. **Address Small Sample Size in Interpretation**
  - **Issue**: With just 40 participants, some results—like "perfect separation" in logistic regression—might be artifacts of overfitting.
  - **Required Action**: Clearly state that **results are preliminary and exploratory**, and that **larger studies are needed** to validate these regression models and confirm predictors.- Added in limitations
3. **Include AUC Curves or Graphs for Sex Prediction**
  - **Issue**: AUC values are reported, but no visual ROC curve or regression diagnostics are shown.
  - **Required Action**: Include **graphical output** of logistic regression model (e.g., ROC curve, classification plot) to visually support claims of predictive strength.- ROC curves are added as Figure 5

�� **Minor Revisions Suggested**

1. **Language and Grammar**
  - Rephrase awkward phrases:
    - “Subjects who do not having ear deformity” → “Subjects without any ear deformities.”- Changed
    - “Measurement was performed twice by two people” → “Each measurement was independently performed twice by two observers.”- Changed
  - Perform a **light grammar and clarity check** across the manuscript.- Done all grammatical mistakes are corrected throughout the manuscript.
2. **Duplicate or Misplaced Text**
  - Table headers or descriptions appear to repeat on some pages (possibly due to PDF formatting).- Removed
  - Check for **repetitive statistical table text** and ensure **clean formatting** before indexing.- Done
3. **Improve Figure Captions and Labels**
  - **Figures 1–4** could use more descriptive captions to be interpretable on their own.- Added more description to all the figures legends.
  - Include **scale markers** where appropriate.- As measurements were not taken on images we can’t show the scale markers. In images we have shown from where-to-where measurements has been taken.

# Reviewer report: Reviewer response for version 2

**DOI:** 10.5256/f1000research.179146.r371386

**Published:** 2025-03-21

**Authors:** Jolly Agarwal

**Affiliations:** 1 ANATOMY, Government doon medical college, Dehradun, Uttarakhand, India

no comments

Is the work clearly and accurately presented and does it cite the current literature?

Partly

If applicable, is the statistical analysis and its interpretation appropriate?

Yes

Are all the source data underlying the results available to ensure full reproducibility?

Yes

Is the study design appropriate and is the work technically sound?

Yes

Are the conclusions drawn adequately supported by the results?

Yes

Are sufficient details of methods and analysis provided to allow replication by others?

Yes

Reviewer Expertise:

Anatomist, Osteology, Medical Education, Histology and Radiology

I confirm that I have read this submission and believe that I have an appropriate level of expertise to confirm that it is of an acceptable scientific standard.

# Reviewer report: Reviewer response for version 1

**DOI:** 10.5256/f1000research.176552.r363729

**Published:** 2025-03-04

**Authors:** Nurdan Sezgin

**Affiliations:** 1 Kütahya Health Sciences University, Kütahya, Turkey

A current issue has been addressed. I suggest updates on a few issues:

 - Not enough current publications have been included

 - Conclusion is not satisfactory enough. I attribute this to the small number of subjects.

 - If the term "population" is in the title, 40 people is an unacceptable number. In this case, I do not see it possible to rewrite the study with new participants. Therefore, the word "population" should be removed from the study title.

Is the work clearly and accurately presented and does it cite the current literature?

Partly

If applicable, is the statistical analysis and its interpretation appropriate?

Yes

Are all the source data underlying the results available to ensure full reproducibility?

Yes

Is the study design appropriate and is the work technically sound?

Yes

Are the conclusions drawn adequately supported by the results?

Partly

Are sufficient details of methods and analysis provided to allow replication by others?

Yes

Reviewer Expertise:

Forensic anthropology, identification, age and sex estimation from facial images and ear images

I confirm that I have read this submission and believe that I have an appropriate level of expertise to confirm that it is of an acceptable scientific standard, however I have significant reservations, as outlined above.

# Response

**Published:** 2025-03-08

**Authors:** Chandni Gupta

**Affiliations:** Anatomy, Kasturba Medical college, Manipal, Manipal, Karnataka, India

A current issue has been addressed. I suggest updates on a few issues:

 - Not enough current publications have been included

**Response:-** Added 3 more new references and compared with the present study.

 - Conclusion is not satisfactory enough. I attribute this to the small number of subjects

**Response:- **I have added few more points in conclusion to make it more informative.

 - If the term "population" is in the title, 40 people is an unacceptable number. In this case, I do not see it possible to rewrite the study with new participants. Therefore, the word "population" should be removed from the study title.

**Response:-** I had removed the word population from the study title.

# Reviewer report: Reviewer response for version 1

**DOI:** 10.5256/f1000research.176552.r363735

**Published:** 2025-02-13

**Authors:** Jolly Agarwal, Anurag Agrawal

**Affiliations:** 1 ANATOMY, Government doon medical college, Dehradun, Uttarakhand, India; 2 Pulmonary Medicine, Government Doon Medical College, Dehradun, Uttarakhand, India

**Summary – **The present observational study focuses on evolution of stature and sexual dimorphism of external ear using various morphometric parameters and morphological features for forensic identification. The external ear can be used to evaluate sex and stature of person from various ear measurements which is very useful for forensic anthropologist.

**Major points – **The total 40 individuals were taken for the study in which 20 were male and 20 were female. The age of individual was 18-25 years. Linear and angular measurements of external ear were taken, various morphological parameters of ear were also noted.

 The most common shape in both male and female was oval. The free ear lobe was most common in both male and female. The most common shape of ear lobe in male was arched while in female triangular. In both male and female, the helix was concave marginal. The most common shape of ear tragus in male is round while in females it was long. Darwin’s tubercle was enlarged in both male and female.

 The various morphometric parameters of right and left side like lobe height, ear inclination angle, anti-helical take off angle, concha mastoid angle was statistically significant.

 The morphological ear length in females of right and left side showed statistically significant difference.

 In males, the ear inclination angle, concha mastoid angle of right and left side showed statistically significant difference.

 Gender wises most of the morphometric parameters of ear were statistically significant, therefore sexual dimorphism can be determined.

 In females significant positive correlation between height and ear inclination angle were found therefore ear parameter can be used as predictor of stature of female.

**Minor points – **Height and any of ear measurements were not statistically significant in males.

 The sample size of the study is less which is mentioned in limitation of study.

Is the work clearly and accurately presented and does it cite the current literature?

Partly

If applicable, is the statistical analysis and its interpretation appropriate?

Yes

Are all the source data underlying the results available to ensure full reproducibility?

Yes

Is the study design appropriate and is the work technically sound?

Yes

Are the conclusions drawn adequately supported by the results?

Yes

Are sufficient details of methods and analysis provided to allow replication by others?

Yes

Reviewer Expertise:

Anatomist, Osteology, Medical Education, Histology and Radiology

We confirm that we have read this submission and believe that we have an appropriate level of expertise to confirm that it is of an acceptable scientific standard.
